# Barriers to HIV testing and characteristics associated with never testing among gay and bisexual men attending sexual health clinics in Sydney

**DOI:** 10.7448/IAS.18.1.20221

**Published:** 2015-08-27

**Authors:** Damian P Conway, Martin Holt, Deborah L Couldwell, Don E Smith, Stephen C Davies, Anna McNulty, Phillip Keen, Philip Cunningham, Rebecca Guy, on behalf of the Sydney Rapid HIV Test Study

**Affiliations:** 1 The Kirby Institute, UNSW Australia, Sydney, Australia; 2 Short Street Sexual Health Centre, St George Hospital, Sydney, Australia; 3 Centre for Social Research in Health, UNSW Australia, Sydney, Australia; 4 Western Sydney Sexual Health Centre, Western Sydney Local Health District, Sydney, Australia; 5 The Marie Bashir Institute for Infectious Diseases and Biosecurity, University of Sydney, Sydney, Australia; 6 Albion Centre, Sydney, Australia; 7 School of Public Health and Community Medicine, UNSW Australia, Sydney, Australia; 8 North Shore Sexual Health Service, Royal North Shore Hospital, Sydney, Australia; 9 Sydney Medical School, University of Sydney, Sydney, Australia; 10 Sydney Sexual Health Centre, Sydney Hospital, Sydney, Australia; 11 St Vincent's Centre for Applied Medical Research, UNSW Australia, Sydney, Australia; 12 NSW State Reference Laboratory for HIV, St Vincent's Hospital, Sydney, Australia

**Keywords:** barriers to HIV testing, never testing, gay and bisexual men, sexual health clinics

## Abstract

**Introduction:**

HIV diagnoses among gay and bisexual men have increased over the past decade in Australia. HIV point-of-care testing (POCT) was introduced in Australia in 2011 as a strategy to increase HIV testing by making the testing process more convenient. We surveyed gay and bisexual men undergoing POCT to assess barriers to HIV testing and characteristics associated with not having previously tested for HIV (never testing).

**Methods:**

During 2011 and 2012, gay and bisexual men who were undergoing POCT at four Sydney sexual health clinics self-completed questionnaires assessing testing history and psychological and structural barriers to HIV testing. Bivariate and multivariate logistic regression was used to assess associations between patient characteristics and never testing.

**Results:**

Of 1093 participants, 981 (89.9%) reported ever testing for HIV and 110 (10.1%) never testing. At least one barrier to testing was reported by 1046 men (95.7%), with only 47 men (4.3%) not reporting any barrier to testing. The most commonly reported barriers to testing were annoyance at having to return for results (30.2%), not having done anything risky (29.6%), stress in waiting for results (28.4%), being afraid of testing positive (27.5%) and having tested recently (23.2%). Never testing was independently associated with being non-gay-identified (adjusted odds ratio [AOR]: 1.9; 95% confidence interval [CI]: 1.1–3.2), being aged less than 25 years (AOR: 2.4; 95% CI: 1.6–3.8), living in a suburb with few gay couples (AOR: 1.9; 95% CI: 1.2–3.0), being afraid of testing HIV-positive (AOR: 1.6; 95% CI: 1.0–2.4), not knowing where to test (AOR: 3.8; 95% CI: 1.3–11.2) and reporting one or no sexual partners in the last six months (AOR: 2.7; 95% CI: 1.2–6.2).

**Conclusions:**

Barriers to HIV testing were commonly reported among the clinic-based gay and bisexual men in this study. Our findings suggest further health promotion and prevention strategies are needed to address the knowledge, attitudes and behavioural factors associated with never testing.

## Introduction

HIV infection remains a major global health issue, with gay, bisexual and other men who have sex with men (GBM) disproportionately affected [1]. In 2012, it was estimated that there were 1.6 million AIDS-related deaths and 35.3 million people living with HIV infection worldwide [2]. In recent years, increasing HIV notification rates and increasing risk behaviours have been reported among GBM (especially younger men) in many countries [3–7]. In 2012 to 2013, HIV notifications in Australia reached similar levels to the peak in the early 1990s and there has been a 9% increase in the rate of HIV diagnoses over the past 10 years [3].

More frequent HIV testing may facilitate earlier diagnosis and timely initiation of antiretroviral therapy, which can benefit both individuals and the wider community [8–11]. Clinical guidelines recommend annual HIV testing for all sexually active GBM with more frequent testing (3 to 6 monthly) for higher risk men, yet 10 to 20% of Australian HIV-infected GBM are unaware of their status and men are not testing for HIV as frequently as recommended [12–16]. Barriers to more frequent HIV testing commonly reported among online and community-based samples of GBM include psychological barriers (such as lack of perceived risk, fear of a positive result and concern about confidentiality) and structural barriers (such as inconvenience, finding the time to test, having to return for results and the cost and location of testing services) [17–21].

In Australia, HIV testing is predominantly available via clinics and generally involves collection of a venous blood sample for testing in a pathology laboratory, with results available in three to seven days. Clinic-based HIV testing is widely available and publicly funded by the Australian federal government via the Medicare system. Patients present their Medicare card to service providers to access free or subsidised health care (there may be out-of-pocket fees to pay if patients access testing via private clinics, including general practice clinics). However, accessing HIV testing via a clinic may not be acceptable to all GBM and, because guidelines recommend frequent testing for men at higher risk of HIV, testing can involve many clinic interactions (such as the patient having to return to the clinic for their results) [13,17]. Providing point-of-care testing (POCT) and other novel approaches to HIV testing with quicker and more convenient provision of results can reduce some structural barriers and increase the acceptability of testing, which may facilitate more frequent testing in high-risk populations [22,23]. In response, HIV POCT was implemented in Australia for the first time in 2011, following revision of the National HIV Testing Policy to support this advance. Initially, POCT was provided at sexual health clinics only as a pilot study to assess the feasibility and acceptability of this approach, and this study was called “The Sydney Rapid HIV Test Study.”

In the context of this study, we assessed the barriers to HIV testing among clinic-based GBM undergoing HIV POCT and the factors associated with never previously testing for HIV (never testing). Having implemented HIV POCT in an effort to increase the acceptability of HIV testing to GBM, we assessed the barriers to HIV testing among these men to inform future efforts to further improve the uptake of testing programmes targeting this population.

## Methods

### Setting

The Sydney Rapid HIV Test Study was conducted in four free-to-access publicly funded sexual health clinics with high caseloads of GBM: two in central and two in suburban Sydney. The study aimed to assess barriers to HIV testing among clinic-based GBM, rapid test performance and the acceptability of POCT to patients and providers. Rapid test performance was compared with the standard of care laboratory serology assays used in this setting and patient and staff acceptability were assessed via surveys. Our data on test performance and the acceptability of HIV POCT to patients and providers have previously been published [24–26].

### Ethical statement

The study was approved by the Human Research Ethics Committees of St. Vincent's Hospital, Darlinghurst, and University of New South Wales, Sydney. Clinicians obtained informed written consent from patients prior to enrolment.

### Study population

Men were eligible to participate if they were aged 18 or more, reported sexual contact with another man and requested HIV testing. The GBM population has the highest HIV prevalence in Australia and accounts for 85% of newly acquired HIV infections [3]. Patients from lower HIV prevalence groups and those known to be HIV-positive were excluded.

### Recruitment

GBM were identified during triage and offered enrolment during consultations if they requested HIV testing. No incentives for study participation were provided. The age and reason for declining or non-enrolment was recorded for eligible men who declined to participate or who were not enrolled by clinic staff. Enrolment to the study involved both the patient survey and HIV POCT.

### Patient survey

From October 2011 to August 2012, all participants were invited to self-complete questionnaires during the clinic visits where they received HIV POCT. The questionnaire was focus tested among GBM during its development. Men participated once only and there was no promotion of the study or HIV POCT during the survey period, either externally to the GBM community or internally within the clinics (to avoid any bias that such promotion may have had on responses to questions regarding the acceptability of POCT in the participant questionnaire; i.e. men who had seen an advertisement and sought out POCT may have had more favourable attitudes to POCT than men simply presenting for any type of HIV testing).

Some questions were adapted from the Gay Community Periodic Survey routine behavioural surveillance questionnaires [27]. The survey assessed patient demographics (sexual identity, age and suburb), sexual risk behaviour with casual and regular male partners, previous HIV testing patterns and barriers to HIV testing (Supplementary File 1). Participants were provided with a series of possible reasons why having a HIV test may be less likely (i.e. barriers to testing) and were asked to indicate which of these reasons, if any, were of importance to them. There was no specific time period provided in the questionnaire for having tested recently, so the interpretation of what was a recent test relied upon the perception of the participant.

### Statistical analysis

We used Australian Census data from 2011 to classify participants according to whether they lived in a suburb with few gay male couples or not using a threshold of 2%, which is the proportion of the Australian male population that identifies as gay [28,29]. We compared the ages of eligible men who were enrolled in the study with those who were not enrolled using the Mann–Whitney test. We then conducted a descriptive analysis of participant characteristics (excluding missing data), with stratifications by age and past HIV testing history.

Pearson's chi-squared and Fisher's exact tests were used to assess associations between patient characteristics (such as age, identity, location, numbers of sexual partners and history of unprotected anal intercourse with casual partners [UAIC]) and two key outcomes (barriers to testing and never testing). The proportions of men reporting UAIC in different age groups were compared with Pearson's chi-squared test to assess any association between risk behaviour and age.

Logistic regression was used to assess associations between never testing and individual patient characteristics (demographics, risk behaviour, and barriers to testing). Variables with a bivariate association of *p*<0.05 were entered into the multivariate analysis (block entry method). Missing data were included to maintain power in the analysis. If the proportion of missing data for variables was greater than 2%, missing data were recoded into a “missing” category (but not the reference category). Adjusted odds ratios (AORs) and 95% confidence intervals (CIs) were presented and *p*-values of less than 0.05 (before rounding) in the multivariate analysis were considered significant.

Analyses were performed using Stata (Release 12, StataCorp LP, College Station, Texas).

## Results

### Patients who were not enrolled or were excluded from analysis

Of 1345 eligible GBM patients at the four sites, 1109 (82.5%) were enrolled, 161 (12.0%) declined and 75 (5.6%) were not enrolled. Among those for whom a reason was recorded, the main reason for eligible patients declining enrolment was patient time constraints (52.0%) and the main reason for patients not being enrolled was clinician time constraints (62.1%). Of 1109 enrolled patients, 16 were excluded because their survey or rapid test results were missing leaving 1093 (98.6%) patients in this analysis. The median age of enrolled men did not differ from those who declined and/or were not enrolled (30 vs. 31 years; *p=*0.12).

### Participant characteristics

Of 1093 participants, 956 (87.9%) were gay-identified, 116 (10.7%) identified as bisexual, and 12 (1.1%) as heterosexual. The median age was 30 years (inter-quartile range 26 to 38) and the majority of men lived in a suburb with few (<2%) gay couples. Ever testing for HIV was reported by 89.9% of men and, of men who had tested before, 72.4% reported testing for HIV in the past year (Table 1). Regarding frequency of testing for HIV, the majority (54.6%) of men tested at least twice yearly, 23.9% annually, and 21.5% less often (including men who had never tested).

**Table 1:** Characteristics of participants

Characteristic	*N* a (%)
Sexual identity	
Gay	956 (87.9)
Bisexual/straight/other	132 (12.1)
Age (years)	
18 to 24	218 (20.2)
25 to 34	491 (45.4)
35 to 44	252 (23.3)
More than 45	120 (11.1)
Lives in a suburb with few gay male couples^b^	
Yes	612 (56.0)
No	481 (44.0)
Ever tested for HIV	
Yes	981 (89.9)
No/don't know	110 (10.1)
When last tested for HIV^c^	
6 months or less ago	543 (55.5)
7 to 12 months ago	165 (16.9)
1 to 2 years ago	188 (19.2)
More than 2 years ago	82 (8.4)
HIV status of regular male partner^d^	
Positive	39 (7.2)
Unknown	134 (24.8)
Negative	368 (68.0)
Describe relationship with regular male partner^d^	
Monogamous	195 (33.1)
Open	395 (67.0)
Number of male sexual partners in last 6 months	
None/one	131 (12.1)
2 to 10 men	645 (59.6)
More than 10 men	306 (28.3)
Type of male sexual partners in last 6 months^e^	
Casual partners only	399 (36.5)
Regular partner(s) only	231 (21.1)
Both casual & regular partners	355 (32.5)
Neither casual nor regular partners	108 (9.9)
Condom use for anal intercourse with male casual partners in last 6 months^f^	
Never/sometimes	379 (39.9)
Always	570 (60.1)

a Men with missing data excluded;

b suburb has less than 2% of couples that are gay men in Australian census 2011;

c men who have never tested before excluded;

d men with no regular male partner excluded;

e men with no male sexual partners in the last 6 months excluded;

f men with no anal intercourse with casual male partners in the last 6 months excluded.

Among the participants, 55.7% reported they currently had sex with a regular male partner, and 71.7% reported they currently had sex with casual male partners. Among men with regular partners, 75.2% reported they knew their partner's HIV status, whereas 24.8% reported it was unknown. Of men with regular male partners, 67.0% reported open relationships with those partners.

Among men who reported anal sex with casual male partners in the last six months, 39.9% reported UAIC with those partners; which varied by age, being highest among men aged 18 to 24 years (47.0%), followed by 44.0% of men aged 35 to 44 years, 39.5% of men aged 45 years or more, and 35.1% of men aged 25 to 34 years (*χ* ^2^=9.5, *p=*0.02). Among men who infrequently tested (either never testing or testing less than annually), but also had anal sex with casual male partners in the last six months, 42.6% reported UAIC.

More than one-quarter of participants (28.3%) reported having more than 10 male sexual partners in the last six months. Among men who infrequently tested, 17.8% had more than 10 male sexual partners in the last six months.

### Barriers to HIV testing overall

At least one barrier to testing was reported by 1046 men (95.7%), with only 47 men (4.3%) not reporting any barrier to testing. The six most commonly reported barriers to HIV testing (reported by more than 20% of participants) were “It's annoying to have to return for results” (30.2%), “I haven't done anything risky” (29.6%), “It's stressful waiting for the test result” (28.4%), “I'm scared of a positive result” (27.5%), “I have been tested recently” (23.2%), and “It's difficult to find the time to be tested” (20.6%) (Table 2).

**Table 2:** Barriers to more frequent HIV testing by testing history

Barrier	Ever testers *N* a (%)	Never testers *N* a (%)	Total *N* a (%)	Test & *p*-value
It's annoying to have to return for results	307 (31.3)	23 (20.5)	330 (30.2)	*χ* ^2^=5.5, *p*=0.02
I haven't done anything risky	292 (29.8)	32 (28.6)	324 (29.6)	*χ* ^2^=0.1, *p*=0.79
It's stressful waiting for the test result	284 (29.0)	26 (23.2)	310 (28.4)	*χ* ^2^=1.6, *p*=0.20
I'm scared of a positive result	255 (26.0)	45 (40.2)	300 (27.5)	*χ* ^2^=10.2, *p*<0.01
I have been tested recently	254 (25.9)	0 (0.0)	254 (23.2)	NA
It's difficult to find the time to be tested	206 (21.0)	19 (17.0)	225 (20.6)	*χ* ^2^=1.0, *p*=0.32
I don't like needles/syringes	88 (9.0)	16 (14.3)	104 (9.5)	*χ* ^2^=3.3, *p*=0.07
I don't like having blood taken for the test	52 (5.3)	12 (10.7)	64 (5.9)	*χ* ^2^=5.3, *p*=0.02
It's difficult to get an appointment	47 (4.8)	2 (1.8)	49 (4.5)	*χ* ^2^=2.1, *p*=0.22^b^
I don't like to show my Medicare card	34 (3.5)	3 (2.7)	37 (3.4)	*χ* ^2^=0.2, *p*=1.00^b^
I don't like having a discussion about testing	25 (2.6)	2 (1.8)	27 (2.5)	*χ* ^2^=0.2, *p*=1.00^b^
It costs too much to get tested	20 (2.0)	3 (2.7)	23 (2.1)	*χ* ^2^=0.2, *p*=0.72^b^
I don't know where to go for a HIV test	14 (1.4)	6 (5.4)	20 (1.8)	*χ*^2^=8.6, *p*<0.01

a Missing data included;

b fisher's exact test. NA=not applicable.

Other less common barriers which accounted for <10% of responses each, included: “I don't like needles,” “I don't like having blood taken,” “It's difficult to get an appointment,” and “I don't like to show my Medicare card” (Table 2). Among men who had engaged in UAIC and men with more than 10 male sexual partners in the last six months, “I haven't done anything risky” was still the third most commonly cited barrier to testing, reported by 26.4 and 24.8% of men in each group, respectively.

### Common barriers to HIV testing

Men in the following groups were more likely to report finding it annoying to return for results as a barrier: ever tested men versus never tested men (31.3% vs. 20.5%) (Table 2) and men who had more than 10 male sexual partners in the last six months versus fewer (35.3% vs. 28.2%) (Supplementary Table 5). Men in the following groups were more likely to report fear of testing positive as a barrier: never tested versus ever tested men (40.2% vs. 26.0%) (Table 2); men aged less than 25 versus older (36.7% vs. 25.1%) (Supplementary Table 2); and men who reported a history of UAIC in the last six months versus no UAIC (31.7% vs. 25.2%) (Supplementary Table 4). Men living in suburbs with 2% or greater gay male couples versus less than 2% were more likely to report having had a recent test as a barrier (26.2% vs. 20.9%) (Supplementary Table 3), and men who had 10 or fewer male sexual partners versus more partners in the last six months were more likely to report not having done anything risky (31.5% vs. 24.8%) as a barrier to testing (Supplementary Table 5).

### Less common barriers to HIV testing

Never tested men were more likely than ever tested men to dislike having blood taken (10.7% vs. 5.3%) and not know where to go to test (5.4% vs. 1.4%) (Table 2); non-gay-identified men were more likely than gay-identified men to report a dislike of having to show their Medicare card (10.2% vs. 2.4%) and dislike of having a discussion about getting tested (5.8% vs. 1.9%) (Supplementary Table 1); and men living in suburbs with fewer than 2% gay male couples were more likely than those living elsewhere to report finding it difficult to get an appointment (6.0% vs. 3.3%) (Supplementary Table 3).

### History of never testing by patient characteristics

Overall, 110 (10.1%) men reported never testing. The proportion who had never tested was significantly higher in the following groups: men aged less than 25 years versus older (20.6% vs. 7.7%; *χ* ^2^=32.0, *p*<0.01); non-gay identified men versus gay-identified (17.5% vs. 9.2%; *χ* ^2^=9.0, *p*<0.01); men living in suburbs with few (<2%) gay couples versus elsewhere (13.6% vs. 6.0%; *χ* ^2^=16.6, *p*<0.01); and men reporting 10 or fewer sexual partners in the last six months versus more than 10 partners (11.9% vs. 5.9%; *χ* ^2^=8.8, *p*<0.01). However, the proportion of men reporting never testing did not vary by recent sexual practices with casual male partners: 10.6% of those who reported UAIC in the last six months had never tested for HIV versus 10.1% among those who did not report UAIC (*χ* ^2^=0.1, *p*=0.81).

### Factors independently associated with never testing

In multivariate analysis, never testing was independently associated with being non-gay-identified, being aged less than 25 years, living in a suburb with few gay couples, being afraid of testing HIV-positive, not knowing where to test and reporting one or no sexual partners in the last six months (Table 3).

**Table 3:** Logistic regression analysis of factors associated with never having tested for HIV^a^

				Bivariate analysis		Multivariate analysis	
					
Variable	Category	Ever testers *N* (%)	Never testers *N* (%)	Odds ratio (95% CI)	*p*	Adjusted odds ratio (95% CI)	*p*
Gay identifying	Yes	868 (88.5)	88 (78.6)	1.0 ref	–	1.0 ref	–
	No	113 (11.5)	24 (21.4)	2.1 (1.3–3.4)	<0.01	1.9 (1.1–3.2)	0.03
Age less than 25 years	Yes	173 (17.6)	45 (40.2)	3.1 (2.1–4.7)	<0.01	2.4 (1.6–3.8)	<0.01
	No	808 (82.4)	67 (59.8)	1.0 ref	–	1.0 ref	–
Lives in a suburb with few gay couples	Yes	529 (53.9)	83 (74.1)	2.5 (1.6–3.8)	<0.01	1.9 (1.2–3.0)	<0.01
	No	452 (46.1)	29 (25.9)	1.0 ref	–	1.0 ref	–
Wanted to know their HIV status	Yes	554 (56.5)	77 (68.8)	1.7 (1.1–2.6)	0.01	1.5 (0.9–2.3)	0.10
	No	427 (43.5)	35 (31.3)	1.0 ref	–	1.0 ref	–
Did something that may have put them at risk of HIV	Yes	269 (27.4)	41 (36.6)	1.5 (1.0–2.3)	0.04	1.3 (0.8–2.0)	0.33
	No	712 (72.6)	71 (63.4)	1.0 ref	–	1.0 ref	–
Afraid of testing HIV-positive	Yes	255 (26.0)	45 (40.2)	1.9 (1.3–2.9)	<0.01	1.6 (1.0–2.4)	0.05^b^
	No	726 (74.0)	67 (59.8)	1.0 ref	–	1.0 ref	–
Doesn't know where to go for HIV test	Yes	14 (1.4)	6 (5.4)	3.9 (1.5–10.4)	<0.01	3.8 (1.3–11.2)	0.01
	No	967 (98.6)	106 (94.6)	1.0 ref	–	1.0 ref	–
Dislikes having blood test taken	Yes	52 (5.3)	12 (10.7)	2.1 (1.1–4.2)	0.02	1.9 (0.9–3.9)	0.08
	No	929 (94.7)	100 (89.3)	1.0 ref	–	1.0 ref	–
Description of relationship with regular male partner	Monogamous	163 (16.6)	32 (28.6)	1.7 (1.1–2.8)	0.03	1.6 (0.8–3.1)	0.19
	Open	367 (37.4)	28 (25.0)	0.7 (0.4–1.1)	0.11	0.9 (0.5–1.4)	0.56
	No regular partner	402 (41.0)	46 (41.1)	1.0 ref	–	1.0 ref	–
	Missing data	49 (5.0)	6 (5.4)	1.1 (0.4–2.6)	0.88	1.2 (0.5–3.3)	0.70
Number of sexual partners in last 6 months	None/one	111 (11.3)	31 (27.7)	4.5 (2.4–8.3)	<0.01	2.7 (1.2–6.2)	0.02
	2 to 10 men	582 (59.3)	63 (56.3)	1.7 (1.0–3.0)	0.05	1.4 (0.8–2.4)	0.32
	≥11 men	288 (29.4)	18 (16.1)	1.0 ref	–	1.0 ref	–
Currently has sex with casual male partner(s)	Yes	691 (70.4)	66 (58.9)	0.6 (0.4–0.9)	0.01	1.3 (0.7–2.4)	0.39
	No	290 (29.6)	46 (41.1)	1.0 ref	–	1.0 ref	–
Uses condoms for anal sex with casual partner(s)	Never/sometimes	339 (34.6)	40 (35.7)	0.5 (0.3–0.8)	0.01	0.7 (0.4–1.5)	0.39
	Always	526 (53.6)	44 (39.3)	0.4 (0.2–0.6)	<0.01	0.7 (0.4–1.3)	0.27
	No AIC/no casual partner	116 (11.8)	28 (25.0)	1.0 ref	–	1.0 ref	–

a Missing data included;

b *p*=0.045 before rounding. CI=confidence interval; ref=reference category; AIC=anal intercourse with casual partners.

## Discussion

We found that there were a number of common barriers to HIV testing among GBM undergoing POCT at the participating clinics. Barriers such as “fear of testing positive” were more pronounced in men who had never tested, younger men, and men who reported UAIC, whereas finding it annoying to return for results was more pronounced in men who had previously tested and men with more sexual partners. We also found that one in ten of the men attending sexual health clinics in our study reported never testing and that never testing was independently associated with being non-gay-identified, being aged less than 25 years, living in a suburb with few gay couples, being afraid of testing HIV-positive, not knowing where to test and reporting one or no sexual partners in the last six months.

Three of the top six barriers to testing were psychological (stress in waiting for results, fear of testing positive and perceiving oneself to be at low risk of HIV) and three were structural (having to return for results, having tested recently, and difficulty finding time to test). These reflect common barriers identified in international research with venue-based and online samples of GBM [17–21]. As the interpretation of what was a recent test in our questionnaire relied upon the perception of the participant, some men may have perceived that they had tested recently and this may have inhibited them from testing more frequently than they had previously.

Three of the most common barriers reported in this study (having to return for results, stress waiting for results and difficulty finding time to test) could be addressed by providing HIV POCT for GBM. For the vast majority of men with non-reactive results, POCT removes the need for return visits and most men report that they find POCT less stressful and anxiety-provoking than conventional testing [30,31]. The majority of GBM report that they prefer convenient and accessible HIV testing with rapid or electronic provision of results, such as POCT, express clinics and community-based or self-testing [23,32,33]. Barriers such as the cost of testing and difficulty getting an appointment reported by Australian community-based GBM may represent difficulties accessing testing in general practice and other private clinics, but these were not commonly reported by our sample of GBM in sexual health clinics [17]. Studies assessing barriers to testing among subjects recruited in general practice clinics often focus on the provider perspective, but patients in primary care have identified lack of perceived risk and fear of stigma and a positive result as factors in delayed HIV testing [34–36].

Overall, our sample of GBM appears somewhat younger than men in community samples but is comparable in terms of reported sexual risk behaviour (reporting more than 10 partners and UAIC in the last six months) [7]. It is concerning that among the youngest age group (18 to 24 years) in our study one-fifth reported never testing for HIV, while nearly half reported UAIC. Higher rates of delayed testing and behavioural risk among younger GBM have been noted in American and Australian research where there has been an increasing trend in UAIC and a decline in recent HIV testing [7,19]. Among men who had never tested or tested less than annually in our study, one-sixth had more than 10 sexual partners and one-third reported UAIC in the last six months. Among men reporting engagement in sex without condoms (or a high number of sexual partners), there may be a mismatch between what is perceived as risky behaviour in official guidelines and what gay men consider risk-taking (“not having done anything risky” was the third most prevalent barrier to testing reported by men who had engaged in UAIC and by men who reported more than 10 sexual partners in the last six months) [21]. If the need for more frequent testing is linked to high-risk behaviours when it is promoted to GBM, caution is needed as men who do not perceive their behaviour as risky may not present for testing, which is an argument for continuing to normalize a routine schedule of testing for all sexually active GBM [12]. Individual perception of being at high risk may fade over time if there have been no adverse consequences from that behaviour.

Lack of perceived risk was the second most common barrier to testing in our sample and it remains an important factor in the late diagnosis of HIV infection [37–40]. The proportion of new HIV cases diagnosed late with CD4 counts of less than 350 cells/mm^3^ among Australian GBM remains high at 34% and these men are at greater risk of AIDS-defining illness, non-AIDS health conditions and death, as well as being at higher risk of transmitting HIV inadvertently to others as their virus is uncontrolled [3,41]. Fear of HIV and/or gay stigma and discrimination, and lack of access to culturally appropriate health care and prevention are also important factors in delaying testing and late HIV diagnosis in GBM [37–40,42]. Despite the high rates of reported behavioural risks in our sample, the effects of HIV optimism and the increased life expectancy among those infected over the last 20 years due to combination HIV therapy, two out of five never previously tested men in our study sufficiently feared a positive HIV test result to inhibit them from testing. This suggests enduring HIV stigma. Our finding that non-gay-identified men are more likely than gay-identified men to report showing their Medicare card and having pre-test discussion as barriers to testing suggests that they are concerned about stigma and how they are perceived by others. HIV testing needs to be more accessible and more acceptable to GBM so that they may test more frequently than they do currently.

Regarding our analysis of never previously tested men, many reported prior HIV risk exposure, but many were inhibited from testing due to fear of a HIV-positive result. The independent associations between never testing and living outside traditional gay suburbs with few gay couples, being non-gay-identified and not knowing where to test suggest a group of men who are not involved in gay social networks or who are not aware of gay-friendly clinics [43,44]. Some of these men may not be comfortable attending gay-friendly services due to concern about being identified as gay [45]. The proportion of gay men living in inner-city Sydney suburbs is much higher than in the geographically dispersed and culturally diverse suburbs where the majority of our sample live [29,46]. Though it was not a commonly reported barrier in our study, not knowing where to test could relate to both the location of testing services and whether men perceive available testing services as culturally appropriate for them [19]. As well as living outside traditional gay suburbs and being more likely to report never testing, non-gay-identifying men have reported similarly high rates of behavioural risk to gay-identified men [44,47]. HIV prevention interventions targeting gay men may not reach men who don't identify as such, so interventions that address the specific needs of non-gay-identified men are needed to motivate these men to test more frequently [45,47].

The main strengths of our study were the large sample of GBM at a relatively high risk of HIV, the high participation rate and the clinic-based setting. This allowed evaluation of why men had presented to the sexual health clinic for testing and what inhibited them from doing so previously. Our evaluation also had some limitations. Our findings may not be generalizable to GBM in other settings, such as men testing via general practice clinics or community-based testing services. Not all GBM are conveniently located to community-based testing sites in the central suburbs of Sydney and barriers to testing among the men attending general practice clinics (where almost half of GBM in the Sydney Gay Community Periodic Survey 2014 had their last HIV test) may differ in terms of cost and access to culturally appropriate testing [48]. We did not record an extended range of demographics (such as ethnicity, education level and income) which limited the associations we could evaluate between participant characteristics and HIV testing. Nevertheless, this study adds to the literature because the clinic-based setting is a rare feature of research on barriers to HIV testing among GBM.

Collaboration between government, service providers, and community-based organisations on enhanced public health responses is required to connect with younger, non-gay-identified men living outside traditional gay suburbs and to establish a regular pattern of frequent HIV testing for all sexually active GBM that does not rely on symptoms or self-perceived risk [12,32,49,50]. Regular frequent testing reduces the interval between HIV tests, facilitates serostatus awareness and timely HIV diagnosis among GBM and may in turn reduce infectiousness and HIV transmission through treatment as prevention [23,49]. Provision of HIV test results by alternative methods such as telephone and text message would also reduce the need for multiple clinic visits to get tested and receive (HIV-negative) results [17,50,51]. Innovative ways of delivering culturally appropriate HIV prevention and testing interventions (such as internet chat-rooms, social networking and smartphone applications) can reach both gay and non-gay identifying GBM, reduce HIV risk behaviours and increase testing rates [45,52–54].

## Conclusions

Barriers to HIV testing were commonly reported among the clinic-based GBM in this study. There is a continuing need to promote HIV testing to younger men, non-gay-identifying men, men who do not perceive themselves to be at risk and those who fear testing so that regular frequent testing is encouraged and normalized.

## Supplementary Material

Barriers to HIV testing and characteristics associated with never testing among gay and bisexual men attending sexual health clinics in Sydney

Barriers to HIV testing and characteristics associated with never testing among gay and bisexual men attending sexual health clinics in Sydney
