# Supplementary material for: Barriers to HIV testing and characteristics associated with never testing among gay and bisexual men attending sexual health clinics in Sydney
Source: J Int AIDS Soc. 2015 Aug 27;18(1):20221. doi: 10.7448/IAS.18.1.20221 (PMC4552862; doi:10.7448/IAS.18.1.20221)
Supplement: Barriers to HIV testing and characteristics associated with never testing among gay and bisexual men attending sexual health clinics in Sydney [file JIAS-18-20221-s001.pdf]

Conway DP et al. Journal of the International AIDS Society 2015, 18:20221.
http://dx.doi.org/10.7448/IAS.18.1.20221

Supplementary File 1 – Questions from participant questionnaire

1. Do you think of yourself as

   [1]☐Gay/homosexual [2]☐Bisexual [3]☐Heterosexual
   [4]☐Other (please specify) ☐

2. How old are you?

   ☐ Years

3. What is your postcode or suburb where you live?

   Postcode ☐ or ☐

4. Have you ever had an HIV test?

   [1]☐Yes [2]☐No [3]☐Don't know/Unsure

5. When did you last test for HIV?

   [1]☐Less than a week ago [2]☐1-4 weeks ago [3]☐1–6 months ago [4]☐7–12 months ago
   [5]☐1–2 years ago [6]☐2–4 years ago [7]☐More than 4 years ago [8]☐Never tested

6. Which of the following best describes your usual frequency of HIV testing?

   [1]☐Every 3 months (or more) [2]☐Twice a year [3]☐Once a year [4]☐Less than once a year
   [5]☐I have never been tested before

7. Why are you having a HIV test today? (choose the most important reasons for you and leave those that do not apply to you blank)

   [1]☐I wanted to know my HIV status
   [2]☐It's part of my regular HIV testing pattern
   [3]☐I did something that may have put me at risk of HIV
   [4]☐I have a new regular sexual partner
   [5]☐My doctor/nurse/counsellor suggested I have the test
   [6]☐I have had symptoms that worried me
   [7]☐My regular sexual partner & I both agreed to get tested
   [8]☐My regular sexual partner has done something risky
   [9]☐I had sex with a HIV-positive partner
   [10]☐I had a condom break when I was having sex
   [11]☐I have taken HIV PEP (PEP medication may prevent HIV infection after unsafe sex)
   [12]☐Other (specify) ☐

8. Which of the following make it **less likely** for you to have a HIV test? (choose the most important reasons for you and leave those that do not apply to you blank)

   [1]☐I am scared of getting a HIV positive test result
   [2]☐I have been tested recently
   [3]☐I haven't done anything to put me at risk of HIV
   [4]☐I don't know where to go for a HIV test
   [5]☐It's difficult to find the time to get tested
   [6]☐It's difficult to get an appointment
   [7]☐It costs too much to get tested
   [8]☐I don't like showing my Medicare card when I get tested
   [9]☐I don't like having a discussion with the doctor/nurse/counsellor about getting tested
   [10]☐I don't like having blood taken for the test
   [11]☐I don't like needles/syringes
   [12]☐It's stressful waiting for the test result
   [13]☐It's annoying to have to return for the test result

9. Do you **currently** have sex with **casual** male partners?

   [1]☐Yes [2]☐No

10. Do you **currently** have sex with a **regular** male partner (or partners)?

   [1]☐Yes [2]☐No

11. If you have a **regular** male partner, do you know the result of his HIV antibody test?

   [1]☐Positive [2]☐I don't know/he hasn't had a test [3]☐Negative [4]☐No regular partner

12. How would you describe your sexual relationship with your current **regular** male partner? (choose one)

   [1]☐I have **no** current regular male partner
   [2]☐We are monogamous – **neither of us** has casual sex
   [3]☐**Both my partner and I** have casual sex with other men
   [4]☐I have casual sex with other men but **my partner doesn't**
   [5]☐**My partner** has casual sex with other men but **I do not**
   [6]☐I have **several regular** male partners

13. How many different men have you had sex with in the last 6 months?

   [1]☐None [2]☐One [3]☐2-5 men [4]☐6-10 men [5]☐11-20 men [6]☐21-50 men [7]☐More than 50 men

14. In the last 6 months, how often did you use <u>condoms for anal sex</u> with **casual** male partners?

   [1]☐Never [2]☐Sometimes [3]☐Always
   [4]☐No anal sex with casual partners in the last 6 months
   [5]☐No casual partners in the last 6 months

15. In the last 6 months, how often did you use <u>condoms for anal sex</u> with your **regular** male partner(s)?

   [1]☐Never [2]☐Sometimes [3]☐Always
   [4]☐No anal sex with regular partners in the last 6 months
   [5]☐No regular partners in the last 6 months
