# Supplementary material for: Barriers to HIV testing and characteristics associated with never testing among gay and bisexual men attending sexual health clinics in Sydney
Source: J Int AIDS Soc. 2015 Aug 27;18(1):20221. doi: 10.7448/IAS.18.1.20221 (PMC4552862; doi:10.7448/IAS.18.1.20221)
Supplement: Barriers to HIV testing and characteristics associated with never testing among gay and bisexual men attending sexual health clinics in Sydney [file JIAS-18-20221-s002.pdf]

Conway DP et al. Journal of the International AIDS Society 2015, 18:20221. http://dx.doi.org/10.7448/IAS.18.1.20221
Supplementary File 2 – Supplementary Tables

Supplementary Table 1. Barriers to more frequent HIV testing by identity

Barrier	Gay-identified N[a] (%)	Non-gay-identified N[a] (%)	Total N[a] (%)	Test & p-value
It's annoying to have to return for results	287 (30.0)	43 (31.4)	330 (30.2)	χ^2=0.1, p=0.75
I haven't done anything risky	279 (29.2)	45 (32.9)	324 (29.6)	χ^2=0.8, p=0.38
It's stressful waiting for the test result	269 (28.1)	41 (29.9)	310 (28.4)	χ^2=0.2, p=0.66
I'm scared of a positive result	255 (26.7)	45 (32.9)	300 (27.5)	χ^2=2.3, p=0.13
I have been tested recently	228 (23.9)	26 (19.0)	254 (23.2)	χ^2=1.6, p=0.21
It's difficult to find the time to be tested	202 (21.1)	23 (16.8)	225 (20.6)	χ^2=1.4, p=0.24
I don't like needles/syringes	91 (9.5)	13 (9.5)	104 (9.5)	χ^2<0.1, p=0.99
I don't like having blood taken for the test	56 (5.9)	8 (5.8)	64 (5.9)	χ^2<0.1, p=0.99
It's difficult to get an appointment	44 (4.6)	5 (3.7)	49 (4.5)	χ^2=0.3, p=0.83[b]
I don't like to show my Medicare card	23 (2.4)	14 (10.2)	37 (3.4)	χ^2=22.4, p<0.01
I don't like having a discussion about testing	19 (1.9)	8 (5.8)	27 (2.5)	χ^2=7.4, p<0.01
It costs too much to get tested	20 (2.1)	3 (2.2)	23 (2.1)	χ^2<0.1, p=1.00[b]
I don't know where to go for a HIV test	16 (1.7)	4 (2.9)	20 (1.8)	χ^2=1.0, p=0.30[b]

[a] Missing data included; [b] Fisher's exact test

Supplementary Table 2. Barriers to more frequent HIV testing by age

Barrier	Age <25 years N[a] (%)	Age ≥25 years N[a] (%)	Total N[a] (%)	Test & p-value
It's annoying to have to return for results	60 (27.5)	270 (30.9)	330 (30.2)	χ^2=0.9, p=0.34
I haven't done anything risky	59 (27.1)	265 (30.3)	324 (29.6)	χ^2=0.9, p=0.35
It's stressful waiting for the test result	68 (31.2)	242 (27.7)	310 (28.4)	χ^2=1.1, p=0.30
I'm scared of a positive result	80 (36.7)	220 (25.1)	300 (27.5)	χ^2=11.7, p<0.01
I have been tested recently	49 (22.5)	205 (23.4)	254 (23.2)	χ^2=0.1, p=0.77
It's difficult to find the time to be tested	46 (21.1)	179 (20.5)	225 (20.6)	χ^2<0.1, p=0.83
I don't like needles/syringes	28 (12.8)	76 (8.7)	104 (9.5)	χ^2=3.5, p=0.06
I don't like having blood taken for the test	15 (6.9)	49 (5.6)	64 (5.9)	χ^2=0.5, p=0.47
It's difficult to get an appointment	6 (2.8)	43 (4.9)	49 (4.5)	χ^2=1.9, p=0.17
I don't like to show my Medicare card	4 (1.8)	33 (3.8)	37 (3.4)	χ^2=2.0, p=0.21[b]
I don't like having a discussion about testing	5 (2.3)	22 (2.5)	27 (2.5)	χ^2<0.1, p=1.00[b]
It costs too much to get tested	1 (0.5)	22 (2.5)	23 (2.1)	χ^2=3.6, p=0.06[b]
I don't know where to go for a HIV test	6 (2.8)	14 (1.6)	20 (1.8)	χ^2=1.3, p=0.26

[a] Missing data included; [b] Fisher's exact test

Supplementary Table 3. Barriers to more frequent HIV testing by location

Barrier	<2% gay couples[a] N[b] (%)	≥2% gay couples[a] N[b] (%)	Total N[b] (%)	Test & p-value
It's annoying to have to return for results	172 (28.1)	158 (32.9)	330 (30.2)	χ^2=2.9, p=0.09
I haven't done anything risky	187 (30.6)	137 (28.5)	324 (29.6)	χ^2=0.6, p=0.46
It's stressful waiting for the test result	164 (26.8)	146 (30.4)	310 (28.4)	χ^2=1.7, p=0.20
I'm scared of a positive result	177 (28.9)	123 (25.6)	300 (27.5)	χ^2=1.5, p=0.22
I have been tested recently	128 (20.9)	126 (26.2)	254 (23.2)	χ^2=4.2, p=0.04
It's difficult to find the time to be tested	119 (19.4)	106 (22.0)	225 (20.6)	χ^2=1.1, p=0.29
I don't like needles/syringes	64 (10.5)	40 (8.3)	104 (9.5)	χ^2=1.4, p=0.23
I don't like having blood taken for the test	37 (6.1)	27 (5.6)	64 (5.9)	χ^2=0.1, p=0.76
It's difficult to get an appointment	20 (3.3)	29 (6.0)	49 (4.5)	χ^2=4.8, p=0.03
I don't like to show my Medicare card	26 (4.3)	11 (2.3)	37 (3.4)	χ^2=3.2, p=0.08
I don't like having a discussion about testing	18 (2.9)	9 (1.9)	27 (2.5)	χ^2=1.3, p=0.26
It costs too much to get tested	12 (2.0)	11 (2.3)	23 (2.1)	χ^2=0.1, p=0.71
I don't know where to go for a HIV test	11 (1.8)	9 (1.9)	20 (1.8)	χ^2<0.1, p=0.93

[a] Proportion of couples in suburb that are gay men; [b] Missing data included

Supplementary Table 4. Barriers to more frequent HIV testing by risk behaviour

Barrier	UAIC N[a] (%)	No UAIC N[a] (%)	Total N[a] (%)	Test & p-value
It's annoying to have to return for results	112 (29.6)	218 (30.5)	330 (30.2)	χ^2=0.1, p=0.74
I haven't done anything risky	100 (26.4)	224 (31.4)	324 (29.6)	χ^2=3.0, p=0.08
It's stressful waiting for the test result	100 (26.4)	210 (29.4)	310 (28.4)	χ^2=1.1, p=0.29
I'm scared of a positive result	120 (31.7)	180 (25.2)	300 (27.5)	χ^2=5.2, p=0.02
I have been tested recently	86 (22.7)	168 (23.5)	254 (23.2)	χ^2=0.1, p=0.76
It's difficult to find the time to be tested	86 (22.7)	139 (19.5)	225 (20.6)	χ^2=1.6, p=0.21
I don't like needles/syringes	40 (10.6)	64 (9.0)	104 (9.5)	χ^2=0.7, p=0.39
I don't like having blood taken for the test	29 (7.5)	35 (4.9)	64 (5.9)	χ^2=3.4, p=0.07
It's difficult to get an appointment	17 (4.5)	32 (4.5)	49 (4.5)	χ^2<0.1, p=1.00
I don't like to show my Medicare card	13 (3.4)	24 (3.4)	37 (3.4)	χ^2<0.1, p=0.95
I don't like having a discussion about testing	11 (2.9)	16 (2.2)	27 (2.5)	χ^2=0.5, p=0.50
It costs too much to get tested	10 (2.6)	13 (1.8)	23 (2.1)	χ^2=0.8, p=0.37
I don't know where to go for a HIV test	7 (1.9)	13 (1.8)	20 (1.8)	χ^2<0.1, p=0.96

[a] Missing data included; UAIC=unprotected anal intercourse with casual male partners in the last 6 months

Supplementary Table 5. Barriers to more frequent HIV testing by partner numbers

Barrier	≤10 partners[a] N[b] (%)	>10 partners[a] N[b] (%)	Total N[b] (%)	Test & p-value
It's annoying to have to return for results	222 (28.2)	108 (35.3)	330 (30.2)	χ^2=5.3, p=0.02
I haven't done anything risky	248 (31.5)	76 (24.8)	324 (29.6)	χ^2=4.7, p=0.03
It's stressful waiting for the test result	219 (27.8)	91 (29.7)	310 (28.4)	χ^2=0.4, p=0.53
I'm scared of a positive result	224 (28.5)	76 (24.8)	300 (27.5)	χ^2=1.5, p=0.23
I have been tested recently	178 (22.6)	76 (24.8)	254 (23.2)	χ^2=0.6, p=0.44
It's difficult to find the time to be tested	153 (19.4)	72 (23.5)	225 (20.6)	χ^2=2.3, p=0.13
I don't like needles/syringes	74 (9.4)	30 (9.8)	104 (9.5)	χ^2<0.1, p=0.84
I don't like having blood taken for the test	48 (6.1)	16 (5.2)	64 (5.9)	χ^2=0.3, p=0.58
It's difficult to get an appointment	34 (4.3)	15 (4.9)	49 (4.5)	χ^2=0.2, p=0.68
I don't like to show my Medicare card	28 (3.6)	9 (2.9)	37 (3.4)	χ^2=0.3, p=0.61
I don't like having a discussion about testing	16 (2.0)	11 (3.6)	27 (2.5)	χ^2=2.2, p=0.14
It costs too much to get tested	19 (2.4)	4 (1.3)	23 (2.1)	χ^2=1.3, p=0.35[c]
I don't know where to go for a HIV test	16 (2.0)	4 (1.3)	20 (1.8)	χ^2=0.7, p=0.62[c]

[a] Number of male sexual partners in the last 6 months; [b] Missing data included; [c] Fisher's exact test
